# Quantifying the profile and progression of impairments, activity, participation, and quality of life in people with Parkinson disease: protocol for a prospective cohort study

**DOI:** 10.1186/1471-2318-9-2

**Published:** 2009-01-20

**Authors:** Meg E Morris, Jennifer J Watts, Robert Iansek, Damien Jolley, Donald Campbell, Anna T Murphy, Clarissa L Martin

**Affiliations:** 1 School of Physiotherapy, The University of Melbourne, Melbourne, VIC 3010, Australia; 2 Centre for Health Economics, Monash University, Melbourne, VIC 3800, Australia; 3 Kingston Centre, Southern Health, Cheltenham, VIC 3192, Australia; 4 Faculty of Medicine, Nursing and Health Sciences, Monash University, Melbourne, VIC 3800, Australia

## Abstract

**Background:**

Despite the finding that Parkinson disease (PD) occurs in more than one in every 1000 people older than 60 years, there have been few attempts to quantify how deficits in impairments, activity, participation, and quality of life progress in this debilitating condition. It is unclear which tools are most appropriate for measuring change over time in PD.

**Methods and design:**

This protocol describes a prospective analysis of changes in impairments, activity, participation, and quality of life over a 12 month period together with an economic analysis of costs associated with PD. One-hundred participants will be included, provided they have idiopathic PD rated I-IV on the modified Hoehn & Yahr (1967) scale and fulfil the inclusion criteria. The study aims to determine which clinical and economic measures best quantify the natural history and progression of PD in a sample of people receiving services from the Victorian Comprehensive Parkinson's Program, Australia. When the data become available, the results will be expressed as baseline scores and changes over 3 months and 12 months for impairment, activity, participation, and quality of life together with a cost analysis.

**Discussion:**

This study has the potential to identify baseline characteristics of PD for different Hoehn & Yahr stages, to determine the influence of disease duration on performance, and to calculate the costs associated with idiopathic PD. Valid clinical and economic measures for quantifying the natural history and progression of PD will also be identified.

**Trial Registration:**

ACTRN12609000008224

## Background

Despite Parkinson disease (PD) affecting more than 4 million people world wide [1], little is known about the rate of disease progression, the costs of medical and allied health management, or which measures best quantify change over an episode of care. This trial protocol presents the methods that will be used to map disease progression in PD, as well as to identify valid clinical and economic measures for quantifying the natural history and progression of this debilitating condition.

Parkinson's disease is a chronic and progressive condition that is usually diagnosed in adults over the age of 60 years. The incidence of PD is estimated to increase from 1.4 percent of those aged 65–75 years to 4.3 percent of people aged over 85 years [2,3]. The rapidly ageing population throughout developed countries means that the number of people with diagnosed PD will quickly increase over the next 20 years. In addition to the impairments, activity limitations and participation restrictions that accompany PD, older adults may experience a range of age-related co-morbidities. Therefore, elderly people with PD can be very prone to complications such as falls, pneumonia and psychosis which can add substantially to the already high social and economic burden of the disease.

A recent literature review of cost of illness studies in Europe relating to PD reported that the annualized direct costs per patient ranged from €5,000 to €10,000 in 2002 [4]. Significant contributors to direct health system costs include medication and medical and allied health services [5-8], with a recent Australian prospective cohort study finding that pharmaceutical expenses contributed approximately 35% of total health care costs [5]. Individuals and their carers also face significant home care costs, and productivity losses [8]. Whilst cost of illness studies indicate disease burden at a population level, this information is only relevant where alternative treatment pathways exist, and comparison then undertaken using cost effectiveness methodologies.

Parkinson disease affects the basal ganglia deep within the brain, leading to a reduction in the size and speed of sequential movements, particularly for motor skills [9]. These activities are normally performed automatically, such as walking, balance, postural control, bed mobility, manipulative tasks, speech, conversation and swallowing [10,11]. The basal ganglia also automate cognitive events, mood and behaviour [12]. Dysfunction in these domains can result in difficulty with driving, shopping, attending to financial matters, conversations, mood and relationships [13]. The disease can eventually involve other brain areas which control bowel, bladder, blood pressure, sleep, mood and cognition. Although no cure exists, treatment is available for the symptoms of PD, particularly for movement disorders.

Medication compensates for the reduced levels of dopamine in the brain and increases the size and speed of automatic movements [14]. Unfortunately because of the progression of the disease, benefits diminish and unwanted movements develop which can further impair activity, participation, and well-being [14,15]. Typically this takes place around 3–5 years after medication is commenced. People can then experience fluctuations in mobility, often unpredictably, making life very difficult and requiring more assistance. For these reasons, some clinicians advocate an inter-professional team approach to the management of the complex array of disorders of movement, cognition and autonomic function [16-19]. Because PD is progressive, management incorporates evaluation of the rate of progression of impairments, activity limitations and participation restrictions [20]. Treatment aims to limit the rate and level of progression to enable people with PD and others in their lives to enjoy the highest possible quality of life [21].

The study will be conducted in Melbourne Australia, the home of the Victorian Comprehensive Parkinson Program (VCPP). The VCPP incorporates several large inter-professional teams, a specialist knowledge base, health professional training in best practice, and long term experience in management of problems experienced by those with PD. The specialist knowledge base includes pharmacological knowledge and the patho-physiology of PD symptoms, manifestations and management. It also includes a specific, evidence-based rehabilitation program which aims to enable people with PD to perform normal movements when these are not possible in the setting of medication failure or ineffectiveness. This rehabilitation program is shared by all disciplines and acts as a common language for the patients irrespective of the team member. The program has a proactive approach to management, identifying problems early and instituting appropriate management. All contact points are managed by expert multi-disciplinary teams. People with PD are empowered to make decisions about their own health care and to be actively involved in management under supervision of relevant team members.

The VCPP is an example of one model of care that embraces this philosophy by providing high quality inpatient, outpatient and home-based services from a specialist inter-professional team of clinicians and clinical researchers. It provides services for more than 1500 patients within a 30 km radius and has 2–4 new referrals per week, providing a unique opportunity for clinical research. The team includes neurologists, physical therapists, occupational therapists, speech pathologists, social workers, psychologists, nurses, dieticians and general medical practitioners. Before service provision models such as this can be comprehensively evaluated, there is a need for preliminary investigations to determine the best measurement tools to quantify therapy outcomes and program costs. Further data are also needed to map disease progression in a sample of people with PD from this service, providing a frame of reference against which inter-disciplinary therapy outcomes can later be evaluated. The study protocol presented in this paper aims to address these needs as the first step in measuring the outcomes and costs of an inter-professional team model for the comprehensive management of people with PD.

Thus the key aims of this initial "proof of concept" investigation are to:

(i) Determine which tests and measurement procedures best capture the multi-dimensional and progressive nature of PD, including clinical effectiveness measures and cost related measures;

(ii) Use this robust test battery that is sensitive to the manifestations of PD to quantify the outcomes of disease progression over a sample period of 12 months in a large sample of people from the VCPP;

(iii) Quantify resource use and costs associated with the management of PD from health system and societal perspectives; and

(iv) Develop a regression model to predict the statistical importance of clinical outcome and quality of life (QoL) measures on cost and health care utilization over a 12-month period.

The key research questions are:

(i) What is the natural history of disease progression in the impairment, activity, participation, well-being, and quality of life domains over a 12 month period in a sample of people receiving inter-disciplinary services from the VCPP?

a. Which field-tested protocols and outcome measures best quantify disease progression for use in future clinical trials of new and/or current interventions for the management of PD?

b. How does quality of life for people with PD change with disease progression?

c. How does caregiver strain change with disease progression?

(ii) What are the costs of care (including informal care, services utilized and events attributable to PD by initial disease state and progression from both a health system and societal perspective) for people with PD receiving care from VCPP?

This involves the establishment of a database to store large volumes of costing and clinical data from multiple sources, and the development of a model to predict the statistical importance of clinical outcome and quality of life measures on cost and health care utilization over a 12-month period for the VCPP.

## Methods and design

### Study design

This is a prospective cohort study comprising primary and secondary collection of clinical, health related quality of life and cost data for people with PD.

### Population

We plan to recruit 100 newly referred persons with PD through the VCPP at participating centres in Melbourne, Australia. Inclusion criteria are a confirmed diagnosis of idiopathic PD, informed consent (including to access Medicare Australia claims data), carer participation in case of cognitive impairment (MMSE<24/30), and ability to attend assessment clinics and to complete questionnaires over a 12-month period. People will be excluded from the study if they are Hoehn & Yahr Stage V, non-ambulatory, or have co-existing neurological conditions which are related to significant motor and cognitive function impairment, are unwilling to provide informed consent, or to participate in a clinical trial.

### Sample size

Given that this is a prospective observational cohort study without formal comparison groups, there is no issue of statistical power to be considered. However, we need sufficient numbers of participants to obtain useful estimates of costs, health care utilization and outcome measures, including quality of life. We propose to enrol a total of 100 eligible participants. The VCPP has an average of 2–4 new PD referrals per week (45 weeks of the year) and, anticipating an approximate rate of 75% of patients consenting to participate, it will be feasible to enrol 100 eligible participants over a 16–24 month interval. It is predicted there will be a 5% attrition rate during the 12 month data collection time period. Precision of estimates derived from a cohort of n = 100 participants will be sufficient for continuous variables (e.g. walking speed, mean step length) confidence interval width will be about 0.14 times the PD population standard deviation (e.g. ± 1.7 m/min for walking speed, ± 0.028 m for stride length); for binary outcomes (e.g. falls) the resulting confidence interval width will be less than ± 7% if the underlying rate is 50% and ± 4% if the rate is less than 10%.

### Sampling strategy

Recruitment will be approximately equal numbers across four categories: (i) PD duration < 1 year; (ii) PD duration 1–5 years; (iii) PD duration 6–10 years; and (iv) PD duration greater than 10 years. The definitions of disease severity will be according to the modified Hoehn & Yahr [22] stages.

### Data collection procedures

After obtaining informed consent from people with PD and their carers, we will record a range of baseline measurements (see Table 1). Participants will be seen by a trained therapist either in their own home, at the Kingston clinic or at a local clinic. They will each be seen three times (baseline, 3 months and 12 months) during the course of their participation in the study. A project officer will also contact each participant on ten occasions (month 1, 2, 4–11) for completion of a monthly questionnaire of health-related events.

**Table 1:** Clinical and health related quality of life measures at baseline, 3 and 12 months

**Clinical Measures**	**Baseline**	**3 months**	**12 months**
UPDRS [26]	X	X	X
Modified Dyskinesia Rating Scale [27]	X	X	X
PDQ-39 [28]	X	X	X
EUROQOL-5D	X	X	X
10 m walking test [13]	X	X	X
Timed Up & Go [29]	X	X	X
Parkinson Disease Fatigue Scale (PDF16)	X	X	X
Geriatric Depression Scale	X	X	X
Neuro-psychiatric inventory of care giver burden	X		X
Motor fluctuation diary for previous 24–48 hours (if relevant)	X	X	X

### Method for health-related costs and utilization data collection

People with PD will be asked to complete a monthly questionnaire of health-related events designed to record visits to health care facilities, any out-of-pocket expenses incurred, medications, and transport to health services during the 12 month follow-up period (refer Table 2). Physical therapist surveyors will be trained in the task of reviewing data obtained from these questionnaires and clarifying information when necessary at the 3-month and 12-month visits. A health economist will work throughout the duration of the program to train, support and oversee the surveyors in the collection of health-care related information.

**Table 2:** Economic data

**Economic Data**	**Baseline**	**12 months**	**Monthly**
Employment status (type, hours)	X	X	
Income change ($)	X	X	
Leisure activities (hours)	X	X	
Government allowances	X	X	
Informal care assistance (hours)	X	X	
Formal carer (type, hours, cost)	X	X	
Home Help (type, hours, cost)	X	X	
Community-based services (type, cost)	X	X	
Hospital admission (reason, transport mode, days)			X
Primary health care services (type, frequency)			X
Non-admitted patient services (type, frequency)			X
Medication (name, dose, frequency)			X

We will seek participants' permission to obtain details of costs associated with visits to any Southern Health facility during the course of the 12-month follow-up. We will also seek permission to obtain Medicare Australia individual claims data relating to medical visits, diagnostic tests and other items included on the Commonwealth Medicare Benefits Schedule. These data are indexed by a unique identification number ("Medicare" number), which we shall obtain for each participant. We shall obtain details of all Medicare claims for the 12 months of active follow-up and for the 12 months prior to enrolment in the cohort.

### Statistical analysis

The principal outcome event rates and changes in outcome measures shall be estimated during the 12 month follow-up interval. Generally, these will need only to be measured as binary outcomes, rate estimates for event data (e.g. falls) and sample means and standard deviations. We shall compute appropriate confidence intervals for outcome measures, changes in outcome measures, and differences between cohort strata. We shall employ random-effects cross-sectional time series models to estimate differences between strata using repeated-measures outcome data. Comprehensive patient level cost and healthcare utilization data can be estimated for each patient. Additional resource use attributable to Parkinson related events can also be determined. Regression analysis will identify the main clinical and socioeconomic predictors of cost and resource use over 12 months in a cohort of people with PD receiving comprehensive care.

### Ethics approval

Ethics approvals were obtained from the Southern Health Human Research Ethics Committee (HREC Number 06107B) and Monash University Standing Committee on Ethics in Research Involving Humans (SCERH No. 2006/728MCC) of Australia.

## Discussion

This trial shall provide a deeper understanding of disease progression over a 12 month period in a large community based sample of people with PD. It shall also generate high quality cost measurements and comprehensive health care utilization data for PD by disease stage. High quality standard deviations for outcome and quality of life and caregiver strain measures will be identified which can be used later to plan intervention studies. An additional result will be a battery of clinical measurement tools which have been field-tested and evaluated with respect to known domains of disability and health, measured variance, precision and clinical utility.

Although there is no comparison intervention for a full economic evaluation, the cost measurements could be incorporated into future planned studies on the costs effectiveness of interventions for PD, including comprehensive strategies such as the VCPP compared with routine care. Cost comparisons can be undertaken between cohorts, where cohorts are determined by disease stage. This analysis will be relevant to future research where interventions are aimed at slowing disease progression.

The data will provide us with information required to select suitable measurement tools so that we can compare the effects of conventional care with a comprehensive model of inter-professional practice. Inter-professional coordinated care models for chronic diseases such as PD are becoming more common with models of care recently established in Europe [18,19,23-25] and North America [17]. Collection of cost, clinical and health related quality of life data in complex settings where a range of health services are provided is a resource intensive exercise. By identifying the tests that are most sensitive to the manifestations of PD and the methods by which these data may be collected is likely to reduce data issues associated with complex interventions in the future. Particularly cost data where a range of data sources from community-based primary health care, specialised multidisciplinary outpatient teams to acute hospital settings are likely to be integrated in a single intervention. Ultimately this will reduce the burden of data collection in a trial context for both participants and researchers.

The care giver strain questionnaire will provide detailed information on the indirect costs and resource use associated with PD. These costs are not often included in economic evaluations for chronic disease and use in future research will enable cost effectiveness studies to take a broader societal perspective as compared to a narrow health system perspective. Knowledge of carer burden may also enable more effective community support services for PD directed to areas of need.

By identifying valid, discriminative and feasible tools to quantify changes over 12 months in people with PD as well as mapping typical progression over a 12 month period, we shall be better placed to compare these comprehensive intervention strategies with routine care and absence of intervention.

## Competing interests

The authors declare that they have no competing interests.

## Authors' contributions

RI and MEM conceived the idea for the study and participated in the design of the study. MEM drafted the manuscript for submission to BMC Geriatrics. JJW conceived the idea for the economic data collection component of the study and assisted in drafting the manuscript for submission to BMC Geriatrics. DJ and DC participated in the study design and will provide data analysis and interpretation. ATM provided project management and assisted with subject recruitment and safety monitoring. CLM assisted in drafting the manuscript for submission to BMC Geriatrics. All the authors have read and approved the manuscript.

## Pre-publication history

The pre-publication history for this paper can be accessed here:


